# Wnt/β‐catenin signaling in brain development and mental disorders: keeping TCF7L2 in mind

**DOI:** 10.1002/1873-3468.13502

**Published:** 2019-06-30

**Authors:** Joanna Bem, Nikola Brożko, Chaitali Chakraborty, Marcin A. Lipiec, Kamil Koziński, Andrzej Nagalski, Łukasz M. Szewczyk, Marta B. Wiśniewska

**Affiliations:** ^1^ Centre of New Technologies University of Warsaw Poland

**Keywords:** beta‐catenin, brain development, habenula, mental disorders, neurogenesis, oligodendrogenesis, postmitotic differentiation, TCF7L2, thalamus, Wnt pathway

## Abstract

Canonical Wnt signaling, which is transduced by β‐catenin and lymphoid enhancer factor 1/T cell‐specific transcription factors (LEF1/TCFs), regulates many aspects of metazoan development and tissue renewal. Although much evidence has associated canonical Wnt/β‐catenin signaling with mood disorders, the mechanistic links are still unknown. Many components of the canonical Wnt pathway are involved in cellular processes that are unrelated to classical canonical Wnt signaling, thus further blurring the picture. The present review critically evaluates the involvement of classical Wnt/β‐catenin signaling in developmental processes that putatively underlie the pathology of mental illnesses. Particular attention is given to the roles of LEF1/TCFs, which have been discussed surprisingly rarely in this context. Highlighting recent discoveries, we propose that alterations in the activity of LEF1/TCFs, and particularly of transcription factor 7‐like 2 (TCF7L2), result in defects previously associated with neuropsychiatric disorders, including imbalances in neurogenesis and oligodendrogenesis, the functional disruption of thalamocortical circuitry and dysfunction of the habenula.

## Abbreviations

**ADHD**, attention‐deficit/hyperactivity disorder

**ASD**, autism spectrum disorder

**BD**, bipolar disorder

**CA**, Cornu Amonis

**DG**, dentate gyrus

**DRD**_**2**_, dopamine D_2_ receptor

**DISC1**, disrupted in schizophrenia 1

**DVL**, Dishevelled

**GSK3α/β**, Glycogen Synthase Kinase 3α or β

**HMG**, high mobility group

**iOLs**, immature premyelinating oligodendrocytes

**IPs**, intermediate progenitors

**iPSCs**, induced pluripotent stem cells

**LRP5/6**, lipoprotein receptor‐related protein 5 or 6

**LEF1/TCFs**, lymphoid enhancer factor 1 and T cell‐specific transcription factors

**MD**, major depression

**mOLs**, myelin‐producing oligodendrocytes

**NDD**, neurodevelopmental disorder

**OPCs**, oligodenrocyte precursor cells

**RG**, radial glia

**SCZ**, schizophrenia

**SNP**, single nucleotide polymorphisms

**SVZ**, subventricular zone

**TCF7L2**, transcription factor 7‐like 2

**VZ**, ventricular zone

### Mental disorders: current view on causes

The present view posits that the causes of mental disorders are multifactorial and involve an interplay between various innate vulnerabilities and biological and social environmental risk factors that eventually manifest as mood swings, social deficits, and alterations of the perception of reality. Accumulating evidence suggests that mental disorders that are diagnosed by psychiatrists as different entities are closely related from a biological point of view. Genetic risk factors overlap across these disorders 1, 2, and common structural changes in the brain and deficits in cognitive circuits are seen despite potentially different etiologies 3, 4. On the other hand, advanced brain imaging and high‐throughput genomic studies of psychiatric patients have provided evidence that each of these major mental disorders can be stratified into different biological subclasses based on specific genetic components and so called endophenotypes, i.e., quantitative biological traits, such as gene expression, anatomical alterations, specific behavior, etc. Endophenotype‐based classifications of disorders and links between endophenotypes and specific sets of genes may provide new opportunities to diagnose psychiatric conditions and provide personalized treatment 5, thus underscoring the need to understand the molecular mechanisms that underlie anatomical and functional brain disruptions in mental disorders. The present review seeks to identify some of these mechanisms.

### Canonical Wnt/β‐catenin signaling and LEF1/TCF transcription factors

The first demonstration of an association between the canonical Wnt pathway and mental disorders was the discovery that the classical mood stabilizer lithium activated β‐catenin by inhibiting GSK3α/β 6, which is a key enzyme in the canonical Wnt pathway 7. Many Wnt pathway genes have been associated with mental illnesses during the last 15 years, further implicating the contribution of Wnt signaling in pathogenesis of these disorders. The present review discusses several possible links between functional alterations of canonical Wnt signaling and endophenotypes that have been associated with mental disorders.

**Figure 1:**
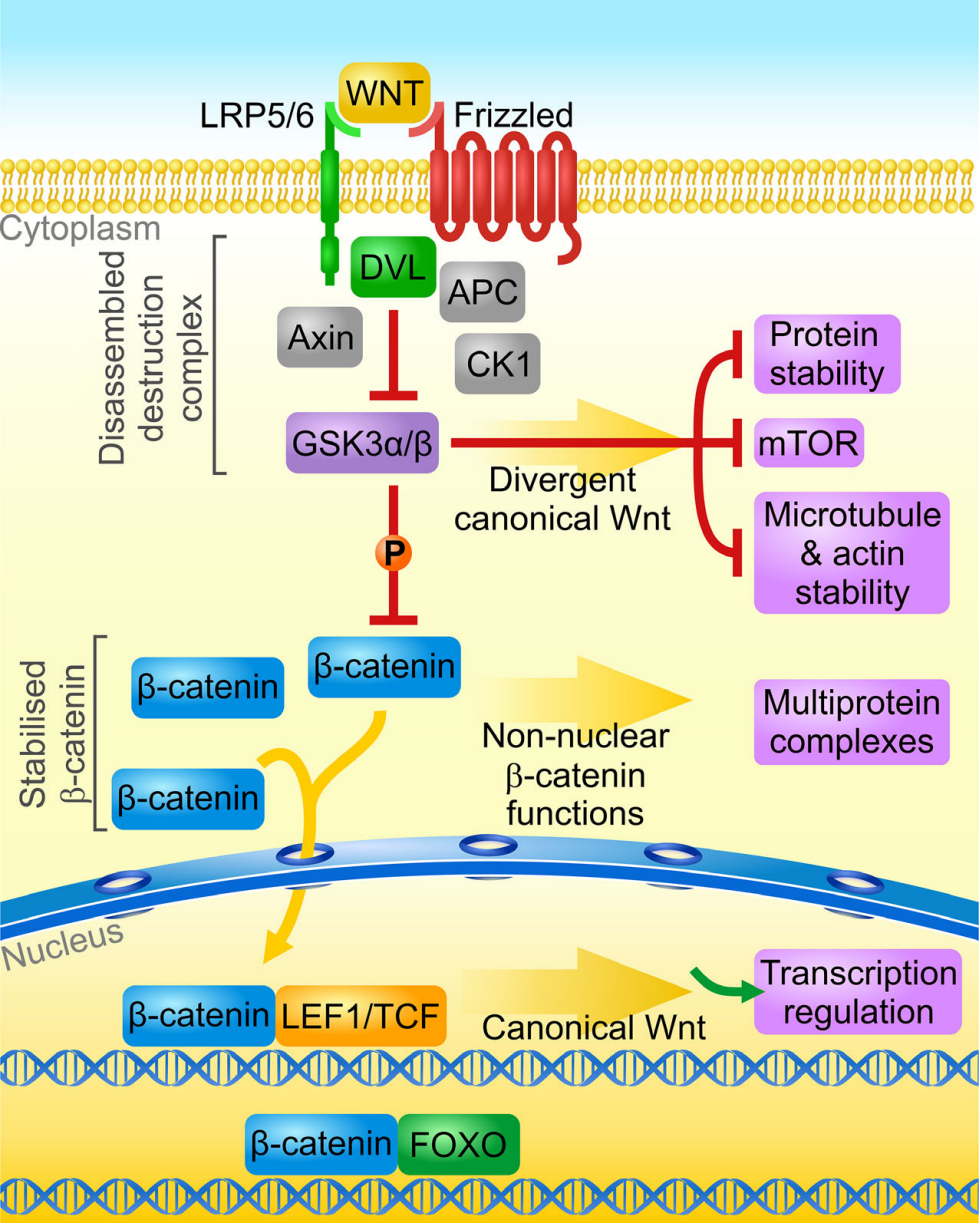
Canonical Wnt/β‐catenin and divergent signaling pathways. The binding of a Wnt ligand to a Frizzled receptor and LRP5/6 co‐receptor, followed by the recruitment of DVL to the receptor complex, leads to translocation and inhibition of the destruction complex, which consists of the kinase GSK3α/β, Adenomatous Polyposis Coli (APC) and Axin. The phosphorylation of β‐catenin by GSK3α/β in the active destruction complex primes this protein for proteasomal degradation (not shown). Upon inhibition of the complex, β‐catenin accumulates in the cytoplasm and translocates into the nucleus where it activates gene transcription as a co‐activator of LEF1/TCFs. This canonical pathway can branch downstream (a) GSK3α/β inhibition (e.g., to slow protein degradation during mitosis, activate the mTOR pathway, or stabilize the cytoskeleton), (b) β‐catenin stabilization (e.g., to stabilize cell adhesion with cadherins or facilitate the assembly of PDZ domain‐containing proteins), and (c) β‐catenin nuclear translocation (to activate gene transcription by interacting with nuclear receptors, FOXO and other transcription factors).

The term ‘canonical Wnt signaling’ has been used as an umbrella term to describe diverse cellular pathways that are activated by Wnt ligands and diverge at the level of Glycogen Synthase Kinase 3α or β (GSK3α/β) inhibition (Fig. 1, Box 1), thus causing ambiguity about the specific pathways to which the authors of various studies refer. Defining the actual pathway is not always straightforward. For example, the terms ‘canonical Wnt’ and ‘Wnt/β‐catenin’ are often used interchangeably to describe a Wnt pathway that is mediated by low‐density lipoprotein receptor‐related protein 5 (LRP5) or LRP6, and GSK3α/β. This can be misleading because one needs to assume that GSK3α/β inhibition in the destruction complex is followed by the stabilization of β‐catenin. In fact, GSK3α/β has many other targets and is involved in many molecular processes. Therefore, the term Wnt/GSK3α/β or upstream canonical Wnt would be more accurate when the downstream effectors are not identified. Even the term ‘Wnt/β‐catenin’ is misleading when considering that the accumulation of β‐catenin is not always followed by its nuclear translocation. The activation of this pathway can also increase other activities of β‐catenin, such as its functions in cell adhesion and protein scaffolding. To help clarify these ambiguities, the present review focuses specifically on the classical canonical Wnt pathway, which is characterized by the translocation of β‐catenin to the nucleus, where it acts as a co‐activator of lymphoid enhancer factor 1 (LEF1)/TCF transcription factors (LEF1, TCF7, TCF7L1, TCF7L2) that bind DNA through the high mobility group domain (HMG). This pathway is further referred to here as the canonical Wnt/β‐catenin pathway.

Box 1 **WNT signaling pathways**

**Figure 2:**
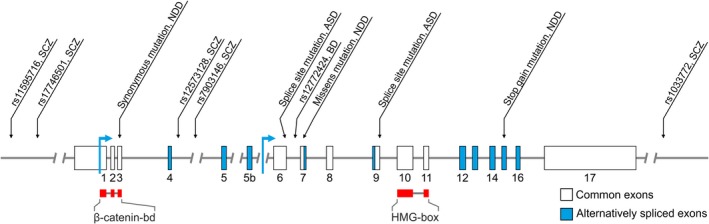
Location of single nucleotide polymorphisms (SNPs) in the human *TCF7L2* gene. Representative intron–exon structure of the *TCF7L2* gene. Long introns are represented by a double slash. Blue boxes indicate the alternatively spliced exons, blue arrows represent alternative transcription start sites. Important protein domains are marked by red boxes: β‐catenin binding domain, and DNA binding domain ‐ HMG‐box. Black arrows indicate the location of SNPs or mutations.

Wnt signaling is traditionally divided into three main branches: the canonical, planar cell polarity (Wnt/PCP) and Wnt/Ca^2+^ 143 pathways. All Wnt signaling pathways are activated by a common mechanism that requires the binding of Wnt‐protein ligands to the cell‐surface receptors Frizzled, and subsequent recruitment of Dishevelled (DVL) proteins. In the canonical Wnt pathway, signaling is transduced by Frizzled receptors together with LRP5 and LRP6 co‐receptors (Fig. 1). This is followed by the disassembly of the so‐called destruction complex, which comprises GSK3α/β and two scaffolding proteins: Axin and Adenomatous Polyposis Coli (APC). At the core of the canonical pathway is inhibition of the GSK3α/β‐mediated phosphorylation of proteins. In the classical view, this leads to the stabilization of β‐catenin (canonical Wnt/β‐catenin pathway), which otherwise is directed to proteasomal degradation upon the phosphorylation by GSK3α/β 7. However, the canonical Wnt pathway can diverge downstream of GSK3α/β to slow down protein degradation in the proteasome (Wnt/STOP pathway), activate mammalian/mechanistic target of rapamycin (mTOR), or stabilize microtubules and the actin cytoskeleton in neurons 144. In the canonical Wnt/β‐catenin pathway, β‐catenin accumulates in the cytoplasm and translocates to the nucleus, where it acts as a transcription co‐activator of LEF1/TCFs 13. Mammals express four members of the LEF1/TCF family (LEF1, TCF7, TCF7L1 which mainly acts as a transcription repressor, and TCF7L2; Fig. 2), which belong to HMG superfamily of transcription factors 145. LEF/TCFs and nuclear β‐catenin can also act independently of each other by interacting with other transcription factors 146, but the majority of β‐catenin binds to chromatin *via* LEF/TCFs 147. β‐catenin, in addition to its function in the nucleus, is involved in multiprotein assembly and cadherin‐mediated cell‐cell adhesion at the membrane. Although the submembranous pool of β‐catenin is largely independent of Wnt signaling, Wnts can sometimes increase the levels of this pool. For example, in neurons, the Wnt/β‐catenin pathway can diverge to modulate dendritogenesis, synaptogenesis, and synaptic vesicle localization independently of nuclear functions of β‐catenin by increasing its interactions with cadherins and PDZ‐containing proteins at the plasma membrane.

Recent reviews have broadly discussed the role of Wnt signaling in the development of pathologies that are associated with mental disorders. However, they focused more either on the role of the divergent GSK3α/β and divergent Wnt/β‐catenin pathways in dendritogenesis, synaptogenesis, and synaptic plasticity, or on upstream Wnt signaling 8, 9, 10, 11, 12, with relatively little coverage of the role of the downstream effectors LEF/TCFs in brain pathologies. We focus on the contribution of these downstream components of the canonical Wnt/β‐catenin pathway and nuclear β‐catenin to the pathogenesis of mental disorders 13. Particular attention is given to the role of TCF7L2, as it has been recently identified as having a central role in neural stem cell differentiation and postmitotic differentiation of some brain regions, impairments of which might contribute to mental disorders.

## Evidence of alterations of canonical Wnt pathway activity and TCF7L2 in mental disorders

### Human genetic studies

The vulnerability to mental illnesses is exacerbated by genetic factors. Although a single causal gene is unlikely to be identified, rates of common polymorphisms and the occurrence of *de novo* mutations in large populations of patients can help to identify molecular pathways that contribute to the pathogenesis of psychiatric disorders 14. Both upstream and downstream elements of the canonical Wnt pathway have been associated with different psychiatric conditions: *WNT1* and *WNT2* with autism spectrum disorder (ASD), *WNT2B* with bipolar disorder (BD), *WNT7A* with ASD and BD, *WNT7B* with schizophrenia (SCZ), *LRP5* with SCZ, attention‐deficit/hyperactivity disorder (ADHD) and major depression (MD), and *LRP6* with ADHD 15, 16, 17, 18, 19, 20, 21. The *CTNNB1* gene, which encodes β‐catenin, has also been associated with ASD and SCZ 16, 22, 23. These examples suggest possible impairments in the canonical Wnt pathway across several major psychiatric disorders.

Among genes that encode downstream nuclear effectors of the canonical Wnt/β‐catenin pathway, only *TCF7L2* has been repeatedly associated with mental disorders in both gene candidate and genome‐wide association studies (Fig. 2). Notably, *TCF7L2*, which is located on chromosome 10 and has been historically denoted T cell‐specific transcription factor 4 (abbreviated as *TCF4*), should not be confused with transcription factor 4 (*TCF4*; located on chromosome 18), which is a SCZ risk factor itself but does not belong to the LEF1/TCF family. Several common intronic polymorphisms and polymorphisms in the proximity of the *TCF7L2* locus have been associated with SCZ 24, 25, 26, 27, 28, 29. Another common variant in an intronic region of *TCF7L2* was identified as a BD susceptibility factor in patients with elevated body mass 30. Some of these polymorphisms (e.g., rs7903146) confer high genetic risk for developing type 2 diabetes, providing a possible molecular explanation for the prevalence of metabolic disorders in SCZ and BP 31. Finally, *de novo* splice site mutations of TCF7L2 were found in ASD patients 32, and stop‐gain and substitution mutations were observed in patients with neurodevelopmental disorders (NDD) 33, 34, 35. These genetic data are a strong indication that TCF7L2 is involved in pathological processes that result in mental disorders. Still unknown, however, are the specific processes because associations of TCF7L2 with specific endophenotypes have not yet been tested. New data from animal studies, discussed below, may shed some light on this issue.

### Animal model studies

Manipulations of individual components of the canonical Wnt pathway in animal models (Table 1) resulted in behavioral changes and provided further evidence of the involvement of this pathway in the pathogenesis of mental disorders. GSK3α/β is the most studied canonical Wnt‐related component with regard to behavioral alterations in animals, because it is a putative target of the classic mood stabilizer lithium. The genetic 36, 37 and chemical 37, 38, 39 downregulation of GSK3α/β (total, or in regions that receive dopaminergic input) decreased the vulnerability to depressive‐like behavior in the forced swim test, tail suspension test or sucrose preference test in mice, whereas *Gsk3b* overexpression in a dopaminergic region 37 had the opposite effect. Other studies implicated low GSK3 activity in mania and psychosis. *Gsk3b* gene knockout in dopamine D_2_ receptor (DRD_2_)‐expressing neurons, which are affected in SCZ, decreased amphetamine‐induced hyperlocomotion and prepulse inhibition, mimicking antipsychotic actions 40. Conversely, the overactivity of GSK3 (by *Gsk3b* overexpression or genetic blocking of inhibitory phosphorylation of GSK3) increased amphetamine‐induced hyperactivity 41, 42, decreased sensorimotor gating 42, 43, and altered the sleep‐wake cycle 44, all of which are considered notable features of mania in BP and psychosis in SCZ. Finally, mice with deficiency in Dishevelled (DVL) activity (by *Dvl1* knockout), which is inhibitory for the GSK3α/β containing destruction complex in the canonical Wnt pathway, exhibited social deficit and stereotyped behavior, reproducing aspects of ASD 43, 45. Because GSK3α/β is a negative regulator of β‐catenin, these studies imply that reducing activity of the Wnt pathway increases the vulnerability to depression, mania, psychosis, and autistic behavior.

The involvement of alterations of GSK3α/β or upstream Wnt signaling activity does not prove the involvement of β‐catenin, not to mention nuclear β‐catenin and LEF1/TCFs. For example, the expression of a stabilized form of β‐catenin that is resistant to GSK3α/β‐mediated degradation (Table 1) did not produce similar antipsychotic effects in aforementioned mice with *Gsk3b* knockout in DRD_2_ neurons, thus excluding such a contribution in this case 40. However, mice with stabilized β‐catenin (in the forebrain and cerebellum or in regions that receive dopaminergic input) exhibited a decrease in the vulnerability to depressive‐like behavior in the forced swim test 46, 47, which was consistent with the aforementioned effects of GSK3α/β inhibition. This suggested the role of β‐catenin in antidepressant‐like response to GSK3α/β inhibition.

The role of LEF1/TCF in behavioral regulation has only recently begun to be investigated. Several experiments tentatively implicated TCF7L2 deficiency in anxiety and depression. The silencing of *Tcf7l2* in zebrafish by morpholinos antagonized the effect of lithium on dark‐induced locomotion 48, suggesting the involvement of TCF7L2 in the behavioral response to this medication for BD. *Tcf7l2* haploinsufficiency increased anxiety‐like phenotypes in the light‐dark box test and contextual fear conditioning 49 and reduced exploratory activity in some mouse strains 50. LEF1 was not shown to be a risk factor in mental disorders. Nevertheless, a recent study found that hypothalamus‐specific *Lef1* knockout increased anxiety‐like behavior in both zebrafish and mice 51, suggesting the possible role of LEF1 in hypothalamic‐pituitary‐adrenal axis activity and the stress response.

Altogether, genetic alterations in individuals with mental disorders and behavioral deficits in genetically modified animals have provided convincing evidence that the canonical Wnt/β‐catenin pathway and LEF1/TCFs (particularly TCF7L2) are involved in the pathogenesis of mental disorders.

## Developmental neurogenesis in the neocortex

### Neocortical abnormalities in mental disorders

The cerebral cortex is the outer layer of the brain. The main part of the cortex in mammals is the neocortex, which processes and integrates emotional and sensory information, controls problem solving and decision‐making. Development of the gyrated human neocortex requires the particularly intensive expansion of neural progenitor populations to produce sufficient numbers of neurons.

Neuroimaging showed changes in cortical thickness and the cortical surface in patients who suffer from psychiatric disorders. These phenotypes may result from excessive or insufficient synaptic pruning or myelination that occurs in adolescents, but also from impaired neurogenesis, e.g., alterations of the ratio of symmetrical cell divisions and asymmetrical cell divisions of neural progenitors. In adult individuals who were diagnosed with MD, a decrease in the thickness of cortical gray matter was observed specifically in the frontal and temporal lobes 52. In patients with BD 53 or SCZ 54, such cortical thinning was more widespread, with the greatest differences in the frontal and temporal lobes. In the ADHD group, a lower volume was also observed predominantly in the frontal cortex, with a reduction of the cortical surface but no changes in cortical thickness 55. By contrast, in the ASD group, the cortical surface was unaffected, but an increase in cortical thickness was observed, especially in the frontal cortex and limbic lobe regions 56. Thus, changes in cortical volume and size are a common endophenotypes in individuals with mental disorders. Unresolved issues include whether such alterations are causes or consequences of the disorder, whether they underlie impairments in neurogenesis, and what are the molecular mechanisms that are involved in these pathologies.

### Canonical Wnt/β‐catenin signaling in the neurogenic niche

Neurons in the neocortex are generated in the ventricular zone (VZ) of the dorsal telencephalon from uncommited radial glia (RG) and in the subventricular zone (SVZ) from neural intermediate progenitors (IPs) 57, 58. Symmetrical cell divisions of RG expand this population, leading to the generation of more ‘radial units’ and consequently surface expansion, whereas asymmetrical cell divisions maintain neural progenitor populations and simultaneously produce neurons that migrate within ‘columnar units’ to generate cortical thickness 59. At later embryogenesis, RG progenitors switch to astrogenic divisions.

The canonical Wnt/β‐catenin signaling pathway is active in the cortical VZ during embryogenesis, as demonstrated by studies in Wnt reporter mice, measurement of *Wnt7a* expression, and the presence of LEF1, TCF7L1, and TCF7L2 60, 61, 62. The activity of Wnt/β‐catenin signaling is high in neurogenic niches but decreases in the intermediate zone that is populated by migrating and differentiating cells 63, 64. Moreover, caudomedially located hem and laterally located antihem, which are two signaling centers of the developing cortex (neocortex and hippocampus), secrete Wnt signals (WNT3A and WNT2B) 65, and Wnt signaling inhibitor SFRP2 (secreted frizzled‐related protein 2 that binds to WNT proteins) 66. This creates a caudomedial to rostrolateral gradient of canonical Wnt/β‐catenin signaling, which is opposite to the gradient of neurogenesis 67. LEF1 expression in the VZ also gradually fades from the medial edge toward the lateral edge, whereas the expression gradient of the transcriptional repressor TCF7L1 is opposite to LEF1 61, 68, presumably contributing to establishment of the gradient. In addition to this spatial pattern, a gradual decrease in Wnt/β‐catenin signaling occurs as development of the cortex progresses 69, 70. Below we discuss how these dynamic spatiotemporal patterns of Wnt/β‐catenin signaling control the timing and progression of neocortical neurogenesis.

### Regulation of proliferation versus differentiation of neural progenitors

Many studies have investigated the role of β‐catenin in different aspects of neurogenesis and neural cell migration. Much effort has been expended to differentiate its function between the regulation of cell adhesion and gene transcription (a focus of the present review), by using genetic modifications in mice (Table 1).

**Table 1:** Wnt pathway genes and genetic modifications mentioned in this review. LOF, loss‐of‐function mutation; GOF, gain‐of‐function mutation.

Mutation	Explanation	Targeted Wnt pathways	Expected effect on the canonical Wnt/β‐catenin pathway
Upstream components of the canonical Wnt pathway			
* Wnt3* overexpression 98	GOF	Canonical and divergent canonical	Upregulation
* Wnt3a* overexpression 75	GOF	Canonical and divergent canonical	Upregulation
* Wnt3a* knockout 65	LOF	Canonical and divergent canonical	Downregulation
* Lrp6* knockout 77, 95	LOF	Canonical and divergent canonical	Downregulation
* Dkk1* overexpression 94, 133	GOF; *Dkk1* encodes an antagonist of LRP5/6	Canonical and divergent canonical	Downregulation
GSK3α/β, a component of β‐catenin destruction complex			
* Gsk3b* knockout 36, 40	LOF	Canonical and divergent canonical	Upregulation
GSK3β K85A/K86A 37	LOF, dominan negative mutant; lysine‐to‐alanine mutations to produce catalytically inactive GSK3β	Canonical and divergent canonical	Upregulation
* Gsk3b* overexpression 37, 42, 44	GOF	Canonical and divergent canonical	Downregulation
GSK3α S21A and GSK3/β S9A 41	GOF; serine‐to‐alanine mutations to block inhibitory serine phosphorylation of GSK3α and β	Canonical and divergent canonical	Downregulation
* *GSK3β S9A 44	GOF; serine‐to‐alanine mutation to block inhibitory serine phosphorylation of GSK3β	Canonical and divergent canonical	Downregulation
Dishevelled (DVL), an inhibitor of β‐catenin destruction complex			
* Dvl1* knockout 43, 45	LOF	Canonical and divergent canonical	Downregulation
β‐catenin (*Ctnnb1*)			
* Ctnnb1* knockout 62, 63, 70, 74	LOF	Canonical Wnt/β‐catenin, divergent Wnt/β‐catenin, Wnt‐independent	Downregulation
* *β‐catenin^*dm*^ (*Ctnnb1* ^*dm*^) 74	LOF, transcriptionally nonfunctional β‐catenin with intact adhesive function; deletions of N‐terminal and C‐terminal transactivation domains	Canonical Wnt/β‐catenin	Downregulation
* *β‐cateninΔexon3 40, 70, 72, 137, 141	GOF, a stabilized form of β‐catenin; deletion of exon3 that encodes the GSK3α/β phosphorylation sites	Canonical Wnt/β‐catenin and divergent Wnt/β‐catenin	Upregulation
* *β‐cateninΔ90 71, 73	GOF, a stabilized form of β‐catenin; deletion of N‐terminal fragment that contains GSK3α/β phosphorylation sites	Canonical Wnt/β‐catenin and divergent Wnt/β‐catenin	Upregulation
* *β‐cateninΔ29–48 75	GOF, a stabilized form of β‐catenin with; deletion of 29–48 aminoacid residues that contain GSK3α/β phosphorylation sites	Canonical Wnt/β‐catenin and divergent Wnt/β‐catenin	Upregulation
* *β‐catenin S37F or S37Y 46, 47	GOF, a stabilized form of β‐catenin; serine‐to‐phenylalanine or serine‐to‐tyrosine mutation in GSK3α/β phosphorylation sites	Canonical Wnt/β‐catenin and divergent Wnt/β‐catenin	Upregulation
* *Constitutively active β‐catenin 70	GOF; β‐catenin activation domain coupled with LEF1	Canonical Wnt/β‐catenin	Upregulation
LEF/TCF transcription factors			
* Tcf7l2* knockout 49, 50, 61, 114, 115, 136, 140	LOF	Canonical Wnt/β‐catenin and Wnt‐independent	Downregulation
* Lef1* knockout 51, 77, 95	LOF	Canonical Wnt/β‐catenin and Wnt‐independent	Downregulation
* Tcf7l1* knockout 61	LOF	Canonical Wnt/β‐catenin and Wnt‐independent	Upregulation
dnLEF1 96, 133	LOF, a dominant negative form of LEF1; LEF1 with a truncated β‐catenin‐binding domain	Canonical Wnt/β‐catenin	Downregulation
dnTCF7L2 63, 69, 78, 96	LOF, a dominant negative form of TCF7L2; TCF7L2 with a truncated β‐catenin‐binding domain	Canonical Wnt/β‐catenin	Downregulation
TCF7L2ΔHMG 139	LOF; TCF7L2 without the DNA binding domain (HMG), likely act as a dominant negative mutant	Canonical Wnt/β‐catenin and Wnt‐independent	Downregulation
LEF1ΔHMG 68	LOF; LEF1 without the DNA binding domain (HMG), likely act as a dominant negative mutant	Canonical Wnt/β‐catenin and Wnt‐independent	Downregulation
* *Dominant‐active LEF1 68, 75, 77, 95	GOF; LEF1 fused to the herpes simplex virus VP16 transactivation domain	Canonical Wnt/β‐catenin and Wnt‐independent	Upregulation

Enhancing canonical Wnt signaling by β‐catenin stabilization has consistently led to the expansion of a progenitor pool that starts at midgestation followed by neocortical hyperplasia 70, 71, 72, 73. Blocking Wnt signaling by *Ctnnb1* knockout had the opposite effect, in which it prematurely decreased the number of proliferating cells in the VZ and led to smaller cortical hemispheres at birth 62. This expansion or reduction of the neocortical neuroepithelium progenitor pool was not attributable to changes in the proliferation rate but rather to alterations of the ratio between cell‐cycle exit and reentry 71, 72. This phenotype was specific for RG, and not for IPs 74, 75, and therefore might be attributable to the cytoskeletal function of β‐catenin because intact apical junctions are critical for maintaining RG polarity and vertical cleavage during mitosis, which result in self‐expanding divisions 58, 76. However, expression of transcriptionally nonfunctional β‐catenin^dm^ with intact adhesive function (*Ctnnb1* ^*dm*^ mutation, β‐catenin allele that lacks N‐ and C‐terminal transcriptional outputs) also resulted in the depletion of progenitor cells and the precocious production of neurons, despite normal VZ architecture 74. Likewise, the focal expression of dominant negative TCF7L2 (dnTCF7L2, lacking β‐catenin binding domain) in the developing neocortex stimulated cell‐cycle exit in progenitors 63, confirming the role of nuclear β‐catenin and LEF1/TCFs in the regulation of neural progenitor pool size in the neocortex.

**Figure 3:**
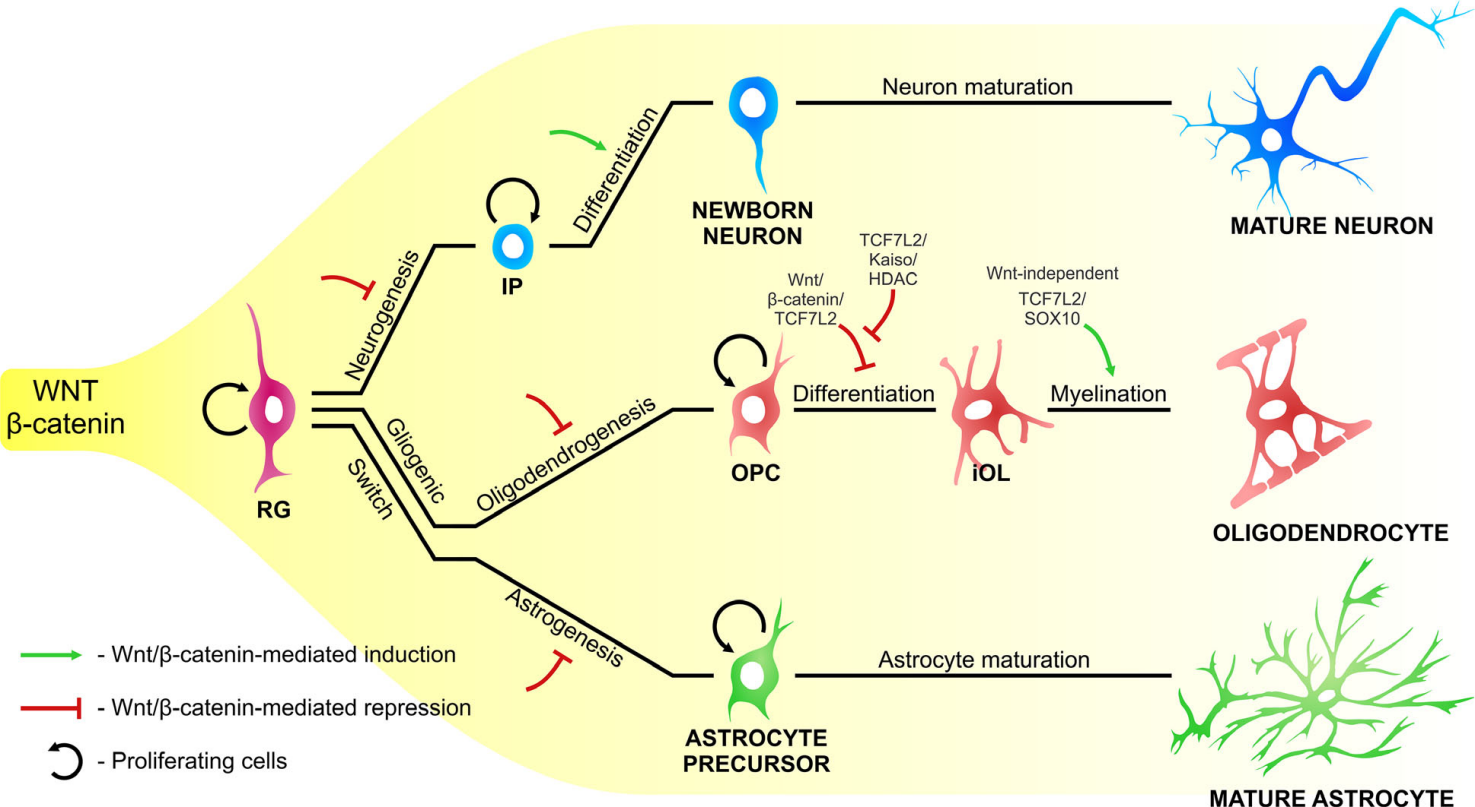
Role of Wnt/β‐catenin signaling and TCF7L2 in neurogenesis and gliogenesis in the neocortex. The process of developmental neurogenesis and gliogenesis in the neocortex is opposite to the temporal gradient of Wnt signaling (shown in yellow). Genetic manipulation to downregulate Wnt/β‐catenin signaling causes premature neurogenic divisions of uncommitted progenitors (RG) and a shorter time of neurogenesis as a consequence of precocious astrogenesis. Wnt/β‐catenin signaling inhibits oligodendrocyte precursor cell (OPC) differentiation. This is antagonized by interactions between TCF7L2 and the Kaiso co‐repressor. TCF7L2 interacts with the SOX10 to promote the further differentiation of iOL into mOL in a β‐catenin‐independent manner. Steps in this process that were shown to be activated or inhibited by Wnt/β‐catenin signaling or TCF7L2 are indicated in green with arrows and red bar‐headed lines, respectively. GP, glial progenitor

Studies in which the uncommitted RG and committed IPs were analyzed as separate populations revealed opposite effects of canonical Wnt/β‐catenin signaling in each population (Fig. 3). In mice with *Lrp6* knockout, *Ctnnb1* knockout or a *Ctnnb1* ^*dm*^ mutation SVZ and postmitotic layer transiently thickened during early neurogenesis, before the depletion of RG finally led to a dramatic decrease in the number of neurons at late neurogenesis or postnatally 62, 74, 77. This was caused by an increase in proliferation of IPs, which are transit‐amplifying cells. Consistent with these findings, the ectopic expression of Wnt3a, dominant active LEF1 (LEF1 fused to the herpes simplex virus VP16 transactivation domain), or stabilized β‐catenin (β‐cateninΔ29‐48) in the developing neocortex promoted the expansion of RG on the one hand, and differentiation of IPs on the other hand 75. This evidence implies a dual role of Wnt/β‐catenin signaling in regulating cell‐cycle mode in uncommitted and committed progenitor populations, in which it maintains RG proliferative (symmetric) divisions and promotes IP differentiative divisions.

A recent study shed some light on which transcription factors of the LEF/TCF family mediate the above effects. Neither LEF1 77, nor TCF7L1 61, appeared to be necessary for development of the neocortex in single knockout studies. Mice with a conditional knockout of *Tcf7l2* exhibited a decrease in progenitor proliferation at late neurogenesis and had a smaller cortex 61, which resembled impairments that were observed in mice with transcriptionally nonfunctional β‐catenin^dm^ 74. This identified TCF7L2 as a regulator of cortical neurogenesis. However, these impairments were already manifested at early and mid‐neurogenesis in *Ctnnb1* ^*dm*^ mutant mice, whereas development of the cortex was unaffected at this stage in *Tcf7l2* knockout mice, and the ratio between RG and IPs was unaltered. This suggests that another transcription factor from the LEF1/TCF family, in addition to TCF7L2, is a mediator of the canonical Wnt/β‐catenin pathway in the developing cortex. *Tcf7l1*/*Tcf7l2* double‐knockout did not deteriorate or rescue the phenotype of single *Tcf7l2* knockout, thus excluding TCF7L1. LEF1 might compensate for the loss of TCF7L2 during neurogenesis in the neocortex, but this hypothesis needs to be tested in *Lef1*/*Tcf7l2* double‐knockout mice.

### Regulation of the neurogenesis timing

Further complicating the role of canonical Wnt signaling in neurogenesis is the involvement of this pathway in the regulation of the timing and duration of neurogenesis. *Lrp6* knockout, *Ctnnb1* knockout, and a *Ctnnb1* ^*dm*^ mutation caused a precocious switch from neurogenesis to astrogenesis in the neocortex 62, 74, 77. Thus, in addition to RG depletion, a shortening of the neurogenic phase could contribute to neocortical hypoplasia in mice with inhibited Wnt/β‐catenin signaling. Moreover, Wnt/β‐catenin signaling possibly regulates the timing of generating successive neuronal populations, which migrate in the neocortical mantle zone in an inside‐out manner, in which later born neurons travel to more superficial parts of the neocortex past early neuron layers 57. The ectopic expression of stabilized β‐catenin starting at mid‐neurogenesis significantly decreased the fraction of late‐born (upper‐layer) neurons 69, 72. Conversely, the fraction of late‐born neurons increased upon the expression of dnTCF7L2 69. *Ctnnb1* knockout and a *Ctnnb1* ^*dm*^ mutation accelerated the production of upper‐layer neurons 74. Overall, these findings suggest that Wnt/β‐catenin signaling prevents a premature switch of neural progenitors to the production of subsequent neuronal populations and regulates the duration of neurogenesis by suppressing the switch from neurogenesis to astrogenesis.

### Regulation of cell migration during neocortical organogenesis

Finally, some studies have investigated the role of nuclear β‐catenin in the establishment of ordered cortical structure. Both the expression of stabilized β‐catenin and *Ctnnb1* knockout in the cortical neuroepithelium led to the early loss of RG apical adherens junctions 62, 70, 74, followed by RG scaffold disassembly 62, 72, a severe migration defect in newborn neurons 62, and the loss of neocortical laminar structure 62, 70, 72, 73, 74, 75. This was likely caused by the loss of β‐catenin function in cell adhesion because polarized architecture of the RG, which is necessary for the radial migration of new neurons, is based on β‐catenin‐mediated interactions between N‐cadherin and the cytoskeleton in apical junctions 76. Indeed, apical junctions and laminar structures were intact during neurogenesis, when canonical Wnt signaling was attenuated downstream of β‐catenin stabilization, by a *Ctnnb1* ^*dm*^ mutation or the knockout of *Lef1*,* Tcf7l1*, or *Tcf7l2* 61, 74, 77. On the other hand, the ectopic expression of *in utero*‐delivered dnTCF7L2 reduced migratory speed in cortical layers, but it did not affect RG scaffolds 78, whereas expression of the β‐catenin activation domain coupled with LEF1 (constitutively active β‐catenin) to activate nuclear β‐catenin signaling, caused cell intermingling between the VZ and SVZ 70. This confirmed the role of canonical Wnt/β‐catenin signaling in some aspects of neuronal migration, but not in the radial organization of migration. Therefore, although integrity of the neuroepithelium during neuronal generation and the initial radial migration of neurons require an intact adhesive function of β‐catenin, maintaining RG assembly and borders between different layers at later stages, as well as new neuron migration along RG basal processes, is at least partly controlled by the nuclear activity of β‐catenin. This is further supported by overrepresentation of cell adhesion genes in putative target genes of LEF/TCFs 79.

### Implications for mental disorders

The association between Wnt signaling, cortical size, and adult behavioral anomalies has been demonstrated in mouse studies. Mice with deficiency in DVL activity (*Dvl1* ^−/−^ mice), in which Wnt signaling was decreased but not entirely abolished, exhibited transient increases in brain weight and cortical thickness at mid‐gestation, accompanied by a reduction of RG and transient expansion of IP population and deep layer neurons. In adulthood, the cortex of these mice was smaller than controls (consistent with RG depletion), but the frontal cortex was thicker 45, resembling the cortical endophenotype of ASD patients. These mice exhibited social deficits and stereotyped behaviors, which were rescued by transient administration of the canonical Wnt agonist CHIR99021 (a selective inhibitor of GSK3β) at mid‐gestation.

Another link is chromodomain helicase DNA‐binding protein 8 (CHD8), a chromatin remodeling factor which regulates Wnt signaling. *De novo* mutations of *CHD8* are one of the most replicated genetic alterations in ASD. *Chd8* knockdown in mice by the *in utero* delivery of shRNA caused the depletion of RG and premature production of neurons during development, and behavioral alterations in adulthood, including social deficits 80. Both developmental and behavioral impairments were rescued by the simultaneous expression of stabilized β‐catenin.

Human induced pluripotent stem cells (iPSCs), *in vitro* cell differentiation, and brain organoid technologies have provided novel opportunities to model SCZ and BD. The causal role of canonical Wnt pathway downregulation was investigated in neural lineage cells that were derived from two related BD patients 81. These cells were characterized by impairments in proliferation and neurogenesis and *WNT7B* downregulation compared to cells derived from healthy relatives. The treatment of these cells with the canonical Wnt agonist CHIR99021 significantly increased LEF1 levels and rescued proliferation deficits. Finally, XAV939, an inhibitor of the canonical Wnt/β‐catenin pathway (a stabilizer of Axin in the β‐catenin destruction complex), rescued morphological impairments in iPSC‐derived cerebral organoids with mutations of the disrupted in schizophrenia 1 (*DISC1*) gene 82, the first gene that was identified as a risk factor for SCZ, BP, and MD. Whether these effects indeed depended on LEF/TCFs was not examined. These findings preliminarily supported the canonical Wnt and neurogenesis hypothesis of mental disorders, but such studies are still too scarce to draw more definitive conclusions.

## Neurogenesis in the hippocampus

### Hippocampal abnormalities and hippocampal neurogenesis in mental disorders

The hippocampus is the main part of the evolutionary old allocortex that is composed of two layers of neurons: granule neurons in the dentate gyrus (DG) and pyramidal neurons in Cornu Amonis (CA) fields. This structure is involved in episodic memory formation and the consolidation of emotional memories. An interesting feature of the hippocampal DG is its ability to produce new neurons throughout life. Adult neurogenesis in the hippocampus is presumed to occur in both humans and rodents and play a role in cognitive plasticity.

Neuroimaging studies have repeatedly reported the loss of hippocampal volume in individuals who are diagnosed with ADHD, BD, SCZ, ASD, and MD 56, 83, 84, 85, 86. However, a large study of MD patients suggested that hippocampal atrophy is a consequence rather than a cause of mental disorders because such atrophy occurred in patients after multiple depressive episodes, whereas people who experienced only a single depressive episode did not present this abnormality 84. Several postmortem studies have shown a lower number of proliferating cells in the hippocampus in deceased SCZ patients 87, but technical issues (low rate of adult neurogenesis, as well as low number and quality of samples) have limited the conclusiveness of these findings. To date, no strong evidence has been reported that hippocampal shrinkage is caused by impairments in developmental or adult neurogenesis.

Despite growing interest in adult hippocampal neurogenesis in the context of mental disorders, its causative role is still controversial. Studies on animals are also inconclusive. For example, neogenin‐deficient mice exhibited depressive‐like behavior 88, whereas cyclin D2‐deficient mice did not exhibit any major behavioral deficits 89, even though adult neurogenesis was compromised in both strains. A number of antipsychotic and antidepressant drugs boosted adult neurogenesis in rodents, but many negative results were also reported 90, 91. Last but not least, some controversy exists about the extent to which adult neurogenesis occurs in humans 92. Some studies concluded that in humans neurogenesis in the hippocampus is negligible after infancy, whereas other studies reported evidence that it does not substantially decline with age.

### Regulation of hippocampus development by canonical Wnt/β‐catenin signaling and LEF1/TCF

In general, canonical Wnt signaling is implicated in similar processes in the hippocampus as in the neocortex; therefore we focus on differences between the two regions. The developing hippocampus is located within direct proximity to the hem, which secretes Wnt ligands 66, 93. The expression of *Lef1* is higher in hippocampal progenitors in both the hippocampal VZ and migratory stream compared with the neocortical VZ 61, 68. Thus, developmental neurogenesis in the hippocampus, particularly in the DG, occurs under conditions of much higher canonical Wnt/β‐catenin sinagling activity than in the neocortex and is mostly regulated by *Lef1*.

Consistent with high activity of the canonical Wnt/β‐catenin pathway, the inhibition of canonical Wnt signaling at the selective level of signal transduction or LEF1/TCFs had more pronounced effects in the hippocampus than in the neocortex. *Wnt3a* ^−/−^ mice with conditional *Ctnnb1* knockout, or mice that express LEF1 without the DNA binding domain (LEF1ΔHMG; likely act as a dominant negative mutant for LEF1/TCFs) the hippocampal VZ was truncated at midgestation, and the hippocampus was virtually absent at the perinatal stage 62, 65, 68, 74. Ectopic expression of *Dkk1*, which encodes a conanical Wnt signaling inhibitor Dickkopf 1 that binds to LRP5/6, *Lrp6* knockout or *Lef1* knockout, which only partially inhibited the canonical Wnt pathway, had less severe effects. Nevertheless, the DG, whose progenitor domain is located in direct vicinity to the hem, was smaller 94, 95. Conversely, canonical Wnt gain‐of‐function mutant mice with stabilized β‐catenin had enlarged and poorly organized hippocampi 62, 73, 96. The specific roles of LEF1, TCF7L2, and TCF7L1 in hippocampal developmental neurogenesis have been investigated in gene knockout studies. In *Lef1* ^−/−^ mice, granule cells were absent in the DG, despite a normal number of proliferating cells in the primary neurogenic niche in the VZ 68, 95. By contrast, CA neurons, which originate from a part of the hippocampal neuroepithelium with lower *Lef1* expression, were normally produced in these mice, suggesting a redundant role for LEF1 and other LEF1/TCF family members in this area, similar to the neocortex. *Tcf7l1* knockout did not result in any hippocampal alterations, but the size of both the granule and pyramidal cell layers of the hippocampus was markedly reduced in *Tcf7l2* knockout mice 61, implying a role for TCF7L2 in the regulation of the expansion of hippocampal progenitor, as well as neocortical progenitors. Altogether, these studies demonstrated that canonical Wnt/β‐catenin signaling, activated by WNT3A and mediated predominantly by LEF1 together with TCF7L2 are indispensable for maintaining proliferating hippocampal progenitors.

### Regulation of adult neurogenesis in the hippocampus by canonical Wnt/β‐catenin signaling

Postnatally, the population of hippocampal progenitors becomes restricted to the subgranular zone of the DG and transforms into adult neural stem cells that continue to divide, although at a much lower rate than in embryos. Adult neurogenesis is not simply an extension of a developmental process into the adulthood because the environment in the postnatal brain is not more neurogenic 97, implying that other mechanisms may be involved in the postnatal regulation of granule cell generation. However, in the adult hippocampus, Wnt/β‐catenin signaling is still active, both in the SGZ and DG, which has been demonstrated in Wnt reporter mice 64, 98. The source of Wnt signaling could be hippocampal astrocytes that reside in the SGZ, which expresses *Wnt3* 98, or cells in the hilus of the DG, which expresses *Wnt7a* 99. The expression of LEF1/TCF7L2 is undetectable or very low in the adult hippocampus 99, 100. Some transient expression of these factors may be found in the SGZ and DG, but high reporter gene activity in these regions in Wnt reporter mice is difficult to explain. Genetic modifications of both upstream and downstream (activating and inhibitory) elements of the canonical Wnt/β‐catenin pathway, including the ectopic expression of *Wnt3* or dominant negative LEF1 (dnLEF1) in the SGZ, confirmed its involvement in adult neurogenesis in the hippocampus 96, 98.

### Implications for mental disorders

Growing evidence implicates canonical Wnt signaling‐associated defects in adult hippocampal neurogenesis in pathogenesis of psychiatric conditions. Nonetheless, much of this evidence is indirect and correlative, and, so far, no solid evidence has linked canonical Wnt‐regulated adult neurogenesis and mental disorders. Many studies reported changes in the expression of the upstream and downstream components of canonical Wnt signaling in the hippocampus of animals that were treated with antidepressant and antipsychotic drugs. For example, chronic administration of selective serotonin reuptake inhibitors increased *Wnt2*, Fz9, *Frzb*,* Lef1*,* Ctnnb1*, and *Dvl1* expression in the hippocampus 101. However, the fact that psychiatric drugs affect the canonical Wnt pathway in the hippocampus does not necessarily mean that this connection is crucial for neurogenesis and the treatment of depression.

The connection between adult hippocampal neurogenesis, the canonical Wnt/β‐catenin pathway, and mental disorders has been demonstrated by investigations of DISC1, which can stabilize β‐catenin through direct GSK3β inhibition. In both the developing and adult DG, DISC1 maintains neural progenitor proliferation 102. The expression of stabilized β‐catenin rescued the impairment of RG proliferation caused by *Disc1* silencing, and the canonical Wnt agonist SB216763 (an inhibitor of GSK3β) normalized depression‐like behavior in these mice. This indicates that DISC1 acts through the canonical Wnt pathway to regulate neurogenesis and modulate mental homeostasis. However, whether the protective role of β‐catenin depends on the rescue of neurogenesis, and whether downstream Wnt signaling (nuclear of β‐catenin and LEF/TCFs) is involved has not been directly tested.

## Postmitotic development of the diencephalon

### Thalamus and habenula in mental disorders

The thalamo‐habenular region is a subcortical part of the brain derived from a common developmental progenitor domain. The thalamus relays sensorimotor information to the cortex, process this information in cortico‐thalamic network, and produce goal‐directed behaviors through cortico–basal ganglia–thalamic circuits 103. The habenula controls reward‐ and aversion‐driven behaviors by connecting the prefrontal cortex, limbic system, and basal ganglia with the brainstem monoaminergic system 104, which is a part of the reward system.

Psychiatric disorders have been repeatedly linked with disturbances in thalamic anatomy and connectivity. Decreases in the volume of the thalamus, as well as reductions of functional connectivity between the thalamus and prefrontal region, with an increase in functional connectivity with the motor cortex are commonly detected in SCZ and BD 85, 86, 105, 106. In ASD, this pattern is partially reversed, with thalamic hypoconnectivity to the prefrontal cortex, temporal lobe, and sensory and motor cortices 107. Unfortunately, neuroimaging data from the habenula are scarce and often do not reach statistical significance, because isolating signal from this small region is difficult. Nevertheless, habenular impairments, and mainly impairments in the lateral habenular part, have been associated with behavioral alterations (e.g., depressive‐like behavior and decreased sensory gating) in animal studies, using lesions or deep stimulation of the habenula 108.

### TCF7L2, LEF1 and nuclear β‐catenin in the postnatal diencephalon

Abundant expression of *Tcf7l2* and *Lef1* was specifically observed during postmitotic development and in adulthood in the thalamus, habenula, and some midbrain structures in vertebrates, including primates 48, 100, 109, 110. These TCF7L2‐positive brain regions exhibited massive nuclear β‐catenin accumulation 48, 100, 111, and high reporter gene activity in Wnt reporter mice 111, 112. This accumulation appears to be independent of the upstream components of the Wnt signaling, but dependent of TCF7L2. In thalamic cells, the silencing of *Tcf7l2* in thalamic neurons prevented β‐catenin from entering the nucleus 48, whereas inhibition of upstream canonical Wnt signaling had no effect on the localization of β‐catenin 113. The role of canonical Wnt/β‐catenin signaling in postmitotic neurons in the thalamo‐habenular domain is little understood compared with its role in cortical neural progenitors.

### TCF7L2 in the postmitotic development and maintenance of thalamo‐habenular regions

**Figure 4:**
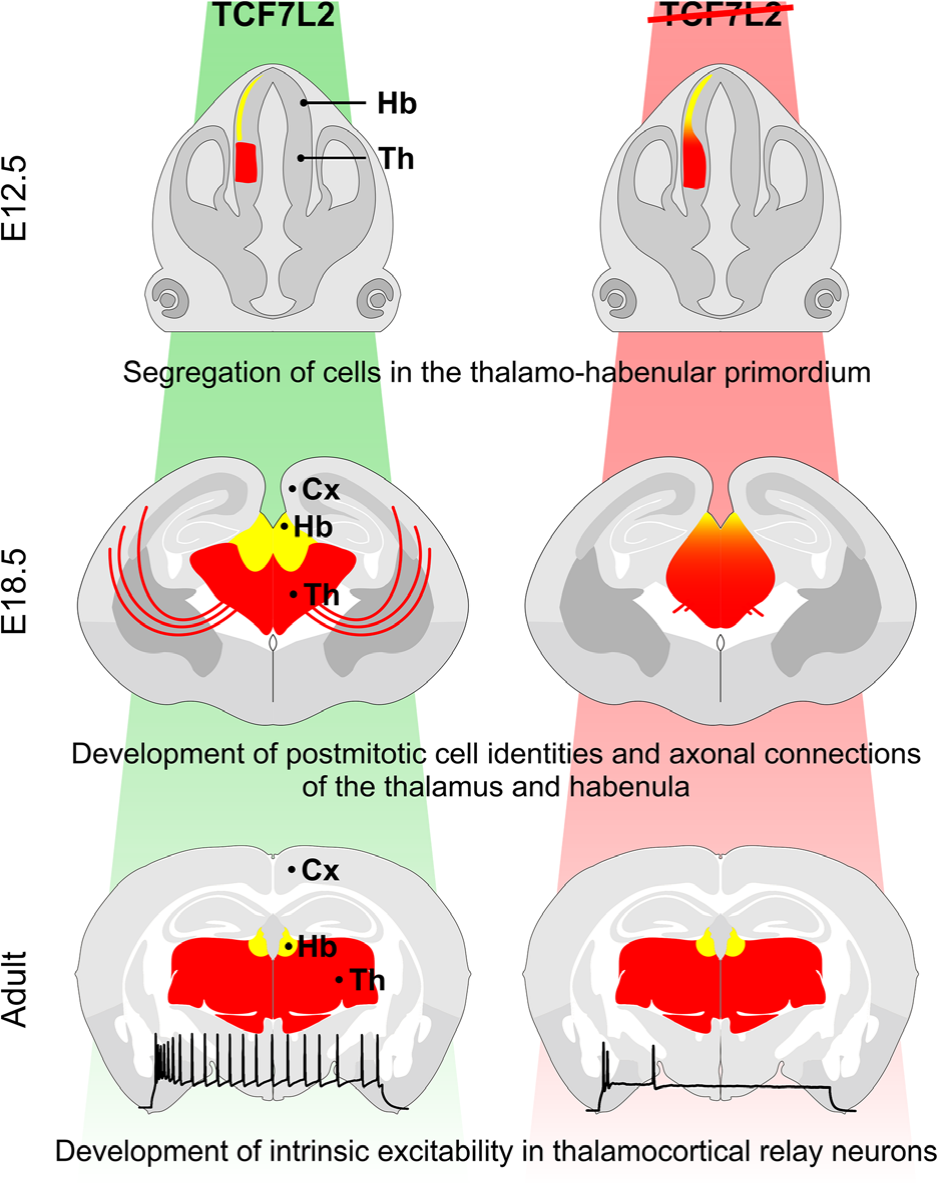
Role of TCF7L2 in the developing thalamus and habenula. Early knockout of *Tcf7l2* impairs segregation of cells in the thalamo‐habenular primordium (upper and middle panel), and disrupts axon growth and regional cell identities (middle panel). Conditional postnatal knockout of *Tcf7l2* impairs intrinsic excitability of thalamo‐cortical relay neurons (bottom panel).

TCF7L2 is not required for initial specification and neurogenesis of the thalamo‐habenular region but its role is important at later developmental stages 114, 115. Recent knockout studies in mice showed a dual role of TCF7L2 in regulating postmitotic development of this region during embryogenesis and postnatally (Fig. 4). During embryogenesis, the lack of TCF7L2 resulted in the loss of anatomical boundaries between the thalamus, habenula, and adjacent structures. The nuclear organization and molecular postmitotic identity of thalamic and habenular neurons was also lost. Furthermore, thalamocortical axons and fasciculus retroflexus, which is the main bundle of habenular efferent axons, were missing. These defects were most likely underlined by decreases in the expression of a number cell adhesion genes and axon‐navigating genes, which were observed in *Tcf7l2*−/− brain, many of them specific for the thalamus and habenula 115. In mice with the postnatal knockout of *Tcf7l2* (thalamus‐specific), the expression of these genes was not altered, but the expression of many genes that are induced in this region postnatally was downregulated, among them ion channel and neurotransmitter genes. These results imply that both postmitotic developmental genetic program and postnatal switch in gene expression in the thalamo‐habenular region are controlled by TCF7L2. Further research should address the mechanism of differential gene expression regulation by TCF7L2 in the two developmental stages.

### Implications for mental disorders

Some thalamic dysfunctions might arise from improper neuronal migration and aberrant thalamocortical circuit development, considering that many risk factor genes for psychiatric diseases are involved in this process. For example, numerous genes that are associated with SCZ and ASD encode axon guidance molecules that belong to the families of SLIT and ROBO, Ephrins and EPH receptors, DCC and UNC5, Semaphorins and Plexins, and Neuregulin 116, 117, 118, all of which are involved in axon guidance. Genes from these families were downregulated in the thalamus in TCF7L2‐deficient mice during embryogenesis, including *Dcc*,* Unc5*,* Robo3*,* Epha3*,* Epha8*,* Slit3*,* Nrp1*, and *Ntng1* 115. Cell adhesion genes (e.g., *Cntn4*,* Cntn6*, and *Reln*), which potentially regulate cell migration and axon outgrowth, are another group that is affected by *Tcf7l2* knockout. All of these genes have been linked with SCZ 119, 120, what shows that TCF7L2 might play a role in the etiology of mental disorders.

Ion channels and neurotransmitter genes, some of which were dysregulated in mice with postnatal *Tcf7l2* knockout, are also often associated with psychiatric disorders. Among ion channels whose expression is altered in the adult TCF7L2‐deficent thalamus is *CACNA1C*, which encodes a Ca^2+^ channel. Mutations of these genes are associated with SCZ disorder with the third highest score in the largest genome wide association study to date 121. Mutations of *KCNIP4* are associated with personality disorders and ASD 122. *CACNA1G* was one of the most downregulated genes in neurons derived from BD patients 81. Expression of the serotonin transporter gene *SLC6A4* has been linked with ASD 123, 124, and was also altered in the adult TCF7L2‐deficent thalamus. Functional connectivity impairments in the TCF7L2‐deficent adult thalamus may also be caused by improper synapse formation within target structures of thalamocortical axons. For example, these mice abnormally express the brain‐derived neurotrophic factor (BDNF) gene, which is also associated with SCZ through its involvement in synapse regulation and plasticity 125.

These multiple genetic overlaps between embryonic and adult TCF7L2‐target genes in the thalamus and psychiatric conditions imply TCF7L2‐mediated thalamic alterations in the pathogenesis of mental disorders. This hypothesis has not been yet directly addressed. Future research should explain the relationship between risk variants of *TCF7L2* and endophenotypes of the thalamus, and more thoroughly investigate the role of this transcription factor in behavior regulation in animal models.

## Developmental oligodendrogenesis in the cortex

### Oligodendrogenesis and myelination in the cortex and mental disorders

Oligodendrocytes are myelinating cells in the vertebrate central nervous system. Their role is to provide trophic support and electric insulation to axons to increase the velocity of nerve signal conduction and maintain axonal integrity. The loss of oligodendroglia and cortical demyelination, followed by the demage of axons are the primary pathology in multiple sclerosis and other leukodystrophies. Slowly accumulating evidence implicates disturbances in oligodendrogenesis and impairments in myelination also in the development of mental disorders. However, when compared to neurogenesis, the possible involvement of oligodendrogenesis in the etiology of psychiatric illnesses has been little investigated. Most of this research has focused on SCZ, but some evidence suggests similar endophenotypes in BP. The levels of oligodendrocyte‐ and myelin‐related transcripts were altered in cortical samples from SCZ 126 and BP 127 patients in postmortem studies. This could simply reflect hypomyelination, which is often observed in SCZ patients 128. Nonetheless, a decrease in the density of oligodendrocytes despite a normal number of oligodenrocyte precursor cells (OPCs) in the prefrontal cortex in SCZ patients 129 suggests that defects in the differentiation of oligodendrocyte lineage cells might be one cause of hypomyelination. This was further corroborated by the lower production of OPCs from iPSCs that were derived from SCZ patient fibroblasts 130. These postmortem and iPSCs studies included a small number of samples; therefore, the statistical power of these findings is low. However, several large‐cohort genomic studies that associated markers of oligodendrocytes (*OLIG2* and *SOX10*) and genes that encode myelin proteins (*MBP*,* PLP1*,* MOG*,* MOBP*,* CNP*, and *MAG*) with SCZ 128, 131 suggested that the differentiation of oligodendroglia might be one of primary impairments in the etiology of this disorder.

### Regulation of cortical oligodendrogenesis by Wnt/β‐catenin signaling and TCF7L2

Oligodendroglia is produced from RG in the VZ/SVZ of the spinal cord and several niches in the brain during embryonic development and postnatally, after neurogenesis is completed. In rodents, most of oligodendrocytes in the adult cortex are derived from the cortical VZ/SVZ of the dorsal telencephalon. OPCs are produced in this niche postnatally and migrate radially into the corpus callosum (a nerve tract beneath the cortex) and cortex. The generation of functional oligodendrocytes occurs through several intermediate stages. Proliferating OPCs generate immature premyelinating oligodendrocytes (iOLs), which finally maturate into myelin‐producing oligodendrocytes (mOLs) (Fig. 3) 132.

Oligodendrocyte precursor cells begin to be produced in the cortical VZ/SVZ when the level of canonical Wnt/β‐catenin signaling activity, which is high during neurogenesis, decreases 133. This downregulation is critical for the switch between neurogenesis and oligodendrogenesis, demonstrated by the loss of OPCs and mOLs after the ectopic expression of stabilized β‐catenin in early oligodendroglia progenitors 134, 135, and the precocious generation of OPCs after the early ablation of *Ctnnb1* in oligodendroglia lineage cells 135. This was further supported by an *in utero* injection of *Dkk1* or a construct that encoded dnLEF1 to downregulate Wnt/β‐catenin signaling, both of which significantly increased the number of OPCs perinatally in the cortex 133. These data demonstrated that the activity of canonical Wnt/β‐catenin signaling prevents premature oligodendrogenesis from RG, similar to the inhibition of premature astrogenesis.

At subsequent stages of oligodendrogenesis that follow the generation of OPCs, the activity of canonical Wnt/β‐catenin signaling was detected in subsets of differentiating and maturating cells 136, 137. Among LEF1/TCFs, only TCF7L2 is present in oligodendroglia lineage cells at higher levels 134, 138. In a single‐cell RNA‐seq study, *Tcf7l2* transcripts were first observed in OPCs, increased to the highest levels in iOLs, and then decreased to lower levels in mOLs 138. Its expression subsequently fades, but reappears during remyelination in animal models of myelin damage both in the spinal cord and corpus callosum 136, 137, 139. However, the pattern of TCF7L2 protein expression and Wnt reporter gene activity little overlapped 136, 137. Studies on genetically modified mice with impaired β‐catenin and TCF7L2 functions in oligodendroglia suggested opposite roles of Wnt/β‐catenin and TCF7L2. In both oligodendroglia‐specific TCF7L2‐deficient mice (with *Tcf7l2* knockout or TCF7L2ΔHMG mutation) and in mice with the ectopic expression of stabilized (β‐cateninΔexon3) in maturing oligodendroglia, myelin did not form properly in the corpus callosum or spinal cord during development and in demyelination lesions 136, 137, 139, 140, 141.

These puzzling findings were resolved by the genome‐wide mapping of TCF7L2 chromatin occupancy at different stages of oligodendrocyte differentiation *in vitro* 139. This analysis identified two factors whose binding regions overlapped with sites that were occupied by TCF7L2 in two developmental stages: Kaiso (a co‐repressor of transcription, whose expression is high in OPCs) and SOX10. Further investigations revealed that Kaiso, together with histone deacetylases HDAC1/2 (which form a repressive complex with Kaiso), blocks the potential interaction between TCF7L2 and β‐catenin during the transition of OPCs into iOLs, resulting in inhibition of the expression of canonical Wnt/β‐catenin target genes 134, 139, 142. Subsequently, during oligodendrocyte maturation, TCF7L2 interacts with SOX10 at regulatory elements of myelination‐associated genes and activates their expression in a Wnt‐independent manner 139. Thus, TCF7L2 plays a dual role in oligodendrogenesis by inhibiting the canonical Wnt/β‐catenin pathway during OPC differentiation and Wnt‐independently regulating myelin genes in oligodendrocytes.

In summary, after the generation of OPCs, further steps of oligodendrogenesis are sequentially regulated by TCF7L2 (Fig. 3). Wnt/β‐catenin signaling must be inhibited by TCF7L2, together with Kaiso and HDAC1/2, to enable the further differentiation of OPCs, followed by the Wnt‐independent interaction between TCF7L2 and SOX10 to induce myelination.

Possible direct links between mental disorders, Wnt, TCF7L2, and oligodendrogenesis have not been investigated. The only indication that such links exist comes from the aforementioned genetic studies that associated mental disorders with *SOX10* and myelin‐associated genes 128, which are directly regulated by TCF7L2 and SOX10.

## Conclusions

Changes in the volume of cortical areas, thalamo‐cortical dysconnectivity, and white matter microstructural alterations are commonly identified endophenotypes in psychiatric research. Evidence from animal studies strongly implicates canonical Wnt/β‐catenin signaling and TCF7L2‐dependent transcription in the development of these impairments, and human genetic studies have found associations between this pathway and major mental disorders. Variants of *TCF7L2* are among the strongest risk factors for diabetes, and TCF7L2 has been extensively studied in the context of peripheral metabolism regulation, but studies on its role in brain development and pathologies have been relatively scarce. Future research on animals with conditional mutations of *Tcf7l2* may help confirm putative links between TCF7L2, brain phenotypes, and behavioral deficits. Identifying relationships between molecular and cellular events, structural and functional alterations in the brain, and behavioral deficits might contribute to the development of personalized treatments for mental disorders.
